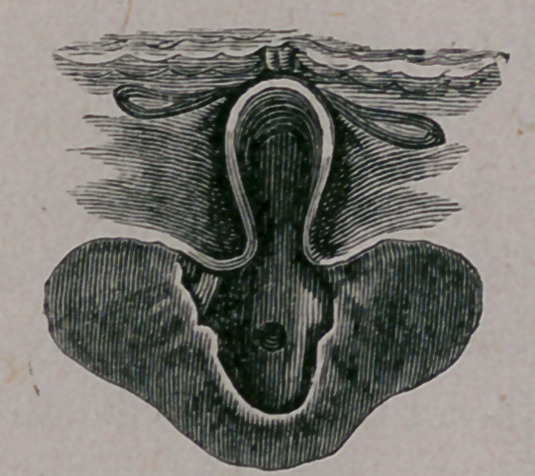# Stricture of the Trachea*Read before the Buffalo Medical Association.

**Published:** 1881-02

**Authors:** Herman Mynter


BUFFALO

### MEDICAL AND SURGICAL JOURNAL.

Vol. XX.—FEBRUARY, 1881.—No. 7.

### Original Communications.

### STRICTURE OF THE TRACHEA.*

BY HERMAN MYNTER, M. D.

Strictures of the larynx have been often met with and described since the invention of the laryngoscope. They are generally of a tuberculous or syphilitic nature. That a simple chronic inflammation of the larynx may produce stricture through hypertrophy of the connective tissue of the under surface of the vocal chords is also a known fact. Ziemssen mentions in his encyclopedia (vol. 4, pages 218-220) several cases of this disease, which he calls chorditis vocalis inferior hyperthrophica. Also in Burow's Laryngoscopical Atlas of 1877, two cases are mentioned and well illustrated. Strictures of the trachea, on the other hand, as a result of a local chronic inflammation, have not been, as far as I know, mentioned in medical literature, and as I have a patient under treatment with this disease, I take the liberty to report the case.

The patient is a married lady, 25 years of age; stirps sana, no specific disease; always enjoyed good health till seven years

* Read before the Buffalo Medical Association.

ago, when she commenced to suffer repeatedly from cough and hoarseness. In course of the next four years these attacks became more frequent, and were more and more difficult to relieve. She consulted several physicians, used all kinds of inhalations, cough syrups and liniments, but was never examined with the laryngoscope until she came to me in October, 1876. She was then very hoarse, and only able to whisper, but stated that she could at times speak with her natural voice for a day or two, but that the slightest cold or irritation immediately made her hoarse again. She was fleshy, and of healthy appearance; the examination of the lungs and heart demonstrated these organs to be perfectly normal at that time. The laryngoscopic examination was impossible, partly on account of the irritability of the mucous membrane of the pharynx, partly, and especially, on account of the deformity and position of the epiglottis, which was bent together from side to side, and stood so obliquely that it prevented any view of the interior of the larynx. Considerable swelling of plicæ aryepiglotticæ and arytænoid cartilages was noted.

Under local treatment with nitrate of silver (gr. 10 to ℥i) and chloride zinc (gr. 20 to ℥i), inhalations of tannin, etc., the swelling gradually diminished, and by daily manipulations and trials with the laryngoscopic mirror and lifting up of the epiglottis with a catheter, the laryngoscopic examination at last became easy. She had in that time used Jod. potash without result, and other medication had also proved futile. The examination showed some congestion of the vocal chords and a partial paralysis of the left recurrent nerve, the chord taking the cadaveric position, especially the adductor muscles being paralyzed. By attempt of phonation crossing of cornicula laryngis was seen; the heart sounds were still normal. At the examination was seen a little red, bleeding spot, just below the anterior commisure. It yielded easily to local treatment with nitrate of silver, but appeared again as often as the treatment was stopped. It had more the appearance of an ulcer with granulating surface than of a tumor.

During the spring of 1877 the cough commenced to be of a convulsive character, and considerable inspiratory dyspnœ occurred during coughing, the expiration being perfectly free. During the following summer it became evident that a stricture was being formed, about one-fourth of an inch below the vocal chords. The whole trachea gradually assumed the same appearance as the spot mentioned, becoming circular and of a deep red granulating character, and easily bleeding. At the same time the heart was found to be somewhat hypertrophied, with severe palpitation, a characteristic aneurismal murmur at its base was heard, and it became evident that there was an aneurism of the arch of the aorta.

Her condition remained the same with the exception, that the stricture gradually contracted more and more, the breathing became more difficult, and in January, 1878, I was obliged to perform tracheotomy, the stricture at that time having contracted so much that its diameter was scarcely more than 5 millimeters. The canula was inserted just below the cricoid cartilage. The respirations became thereafter free, and in a short time the congestion of the vocal chords diappeared, she regained her natural voice, but the paralysis did not improve. The deep-red appearance of the stricture gradually gave place to a whitish shining color; her general health improved, and she complained only of palpitations of the heart. My intention had been to try to dilate the stricture through the canula, possibly by aid of electrolysis, which method I have used with success in two cases of stricture of the œsophagus, but every trial failed. I tried to dilate the stricture per vias naturales, but without success. Through the stricture, which still was contracting, I saw a little whitish prominence, possibly a new growth, below the stricture. Laryngoscopy through the canula was impossible. I have tried it with canulas of all forms and without canulas, but I have never been able to see the under surface of the vocal chords. On July 4th she lost her voice entirely and since has been almost speechless. The examination showed that the stricture had closed up, forming a complete diaphragm and with only a little orifice, as large as the head of a pin, almost in the center, filled generally with mucous. About the same time bleedings commenced through the canula, especially after attacks of coughing, and they have continued daily. Attempts to incise the stricture with a concealed knife were often made, but the wounds thus made healed. Her health has since been very good, with one exception; one year ago last Christmas I was called, and found her in the greatest distress from rapidly increasing dyspnœa. She stated that something was obstructing the right bronchia. I considered it simply the result of pressure from the aneurism, especially as respiration was heard over both lungs, but as she grew rapidly worse and evidently was in extremis, I introduced into the trachea a long pair of forceps. Dr. Hopkins, who kindly visited the patient, concurred with me in the opinion, that her life would terminate in a few hours. With the forceps I got hold of something and stimulated her to cough; the patient was cyanotic in the face and with pulse 160; she coughed up the unknown substance to the fistula, and I removed it with difficulty; it proved to be a perfect cast of the right bronchia, consisting of a solid mass of dried blood, two inches long, and of firm consistency; the breathing became immediately easy and the danger was over.

Her condition now is generally good; she looks healthy, breathes with ease through the canula and takes exercise in the fresh air every day.

She complains of severe palpitation of the heart, and of bleeding through the canula. Examination of heart shows considerable enlargement, the apex being about one inch outside the papillary line. A soft systolic murmur is occasionally heard over the corpus sterni. The canula is pulsating synchronic with the pulse, and I can with the greatest ease count the heart beats by observing the canula. The bleeding occurs every day and seems to be the result of coughing, this occurring first and being followed by the bleeding. I have been unable to find out the source of this bleeding, but my opinion is, that it comes from the space between the stricture and the canula, possibly from some small granulating tumors. It is not easy to determine the source of the hemorrhage. Having first removed the canula and then inspecting the tracheal fistula, something abnormal seems to have taken place; the upper wall of the fistula is seen abnormally long, being about one and a half inches in length, and extends obliquely downwards toward the posterior wall of the trachea, which it almost touches, and leaving but a very small space, which is generally filled with dried clotted blood. It seems, in short, that the anterior wall of the trachea has been pressed backwards by the canula. Her lungs are perfectly normal, and a bleeding from an aneurism could not possibly take place every day for years.

By laryngoscopic examination the epiglottis is seen bent together from side to side, but a view of the larynx is easily obtained; the right vocal chord is seen to move synchronic with inspiration and expiration, even when the little opening in the membrane is filled with mucous; the left vocal chord is in a state of paresis, the adductor muscles being affected, and by phonation the right arytenoid cartilage crosses a little in front of the left. The color of the chords, as far as can be seen on account of the epiglottis, and of the mucous membrane, is almost normal. Below the vocal chord is found a complete diaphragm, having a rather pale color as if consisting of fibrous tissue, and a little hole in the center, generally filled with mucous. The diaphragm has absolutely nothing to do with the vocal chords, seems to be oblique, reaching anteriorly a little higher up than posteriorly, in front reaching almost up to commisura anterior, while behind there is a considerable space between the processus vocales on the one side, and the upper surface of the diaphragm on the other side. I mention this on account of the diagnosis. I feel sure, that the stricture is not the result of a chorditis vocalis inferior, which was the opinion of Drs. Johnston and Hartman, of Baltimore, who saw the patient in my office during the session of the American Medical Association in Buffalo, two years ago.

It seems to me, that, in that case the diaphragm ought to be a direct continuation of the vocal chords. But such is not the case. There is here a considerable space between the vocal chords and the diaphragm, especially behind. Besides, if we had a chorditis vocalis inferior, her voice could scarcely have returned in its normal strength shortly after the tracheotomy and continued so until the stricture closed up. Furthermore, the vocal chords move naturally during inspiration and expiration, which could not be the case if they, so to speak, were glued together by a fibrous tissue. Finally, the perfect ring-form of the stricture from its commencement till now, it seems to me, indicates, that it is formed in an uniformly hollow tube like the trachea, and not in an irregularly formed organ like the larynx. At any rate, illustrations of strictures of the larynx, from any cause, if they are reliable, never show such a ring-formed contraction. I therefore believe, that we have here a genuine stricture of the trachea itself, produced possibly by cicatrization of a ring-formed ulcer. Dr. Seiler, of Philadelphia, who lately examined the patient, has the same opinion. In the literature, which has been at my command, I have not found any such disease in the trachea, except in *Berliner Klinischer Wochenschrift*, of September 9th, 1878, (No. 36). Professor Voltolini, in Breslau, mentions a case of stenosis of the trachea which, is in many regards similar to my case, the greatest difference being that he cured his case by galvanocautery per vias naturales. I will quote his words: "On account of the glottis being wide open, the operation was performed with ease by aid of a common petroleum lamp, before which a biconvex lens was placed. I established, so to speak, a colossal fire in larynx and trachea of the patient, burning into the stenosis not only with the smallest burner but even with the largest in my possession. As the battery was freshly filled, the large instrument quickly became white hot. Having operated at least one hour and a quarter (of course with necessary intermissions on account of the patient), I discovered that the stenosis was not confined to a small space, but consisted in a constriction of the whole trachea." I acknowledge that I do not see how it was possible, before tracheotomy had been performed and without chloroform, to operate in the way mentioned, A treatment like this would in my case have been more difficult, or even impossible, on account of the deformity of the epiglottis and the narrow space to operate in. For me it has always been evident, that nothing could be accomplished per vias naturales.

I have, since the tracheotomy, intended to make a second operation, opening the trachea and larynx from the fistula upwards, and by aid of knife and galvanocautery extirpate the stricture and granulating tumors I might find there, but I have so far been prevented in doing so, partly on account of her heart, as I consider anæsthesia very dangerous, partly on account of the opinion of Drs. Elfsberg and Lefferts, in New York, with whom I have corresponded, and who advised to let the patient alone. She is not at all satisfied with this decision, and is willing to submit to an operation, if she only can regain her voice, and should her heart get better, I am strongly inclined to operate.

It may become necessary on account of the bleeding, which she describes as simply fearful. But besides the bleeding and the palpitations, she is feeling so well at present that she does not feel inclined to run the risk of operative interference. For the cardiac lesion, I have tried every remedy recommended, but without success, and opium alone in two-grain doses relieves her, when the palpitations are very severe. The case offers several interesting features from a laryngoscopic point of view, the deformity of the epiglottis, the trouble commencing with a paresis of left recurrent nerve, and about a year afterwards aneurism of the heart occurring, the stricture of the trachea, occurring below the vocal chords, the abnormal condition of the upper wall of the fistula, etc., and for these reasons I submit it for your consideration.

## Figures and Tables

**Figure f1:**